# Context, mechanisms and outcomes in end-of-life care for people with advanced dementia: family carers perspective

**DOI:** 10.1186/s12904-019-0467-9

**Published:** 2019-10-24

**Authors:** Nuriye Kupeli, Elizabeth L. Sampson, Gerard Leavey, Jane Harrington, Sarah Davis, Bridget Candy, Michael King, Irwin Nazareth, Louise Jones, Kirsten Moore

**Affiliations:** 1 Marie Curie Palliative Care Research Department, Division of Psychiatry, University College London, 6th Floor, Maple House, 149 Tottenham Court Road, London, W1T 7NF UK; 2 Barnet Enfield and Haringey Mental Health Trust Liaison Psychiatry Team, North Middlesex University Hospital, London, UK; 3 Bamford Centre for Mental Health & Wellbeing, University of Ulster, Londonderry, UK; 4 Division of Psychiatry, University College London, London, UK; 5 Department of Primary Care & Population Health, University College London, London, UK

**Keywords:** Dementia, Family carers, Palliative care, Qualitative research, Realist framework, Care homes

## Abstract

**Background:**

Keeping people living with advanced dementia in their usual place of residence is becoming a key governmental goal but to achieve this, family carers and health care professionals must negotiate how to provide optimal care. Previously, we reported a realist analysis of the health care professional perspective. Here, we report on family carer perspectives. We aimed to understand the similarities and differences between the two perspectives, gain insights into how the interdependent roles of family carers and HCPs can be optimised, and make recommendations for policy and practice.

**Method:**

Qualitative study using a realist approach in which we used the criteria from guidance on optimal palliative care in advanced dementia to examine key contexts, mechanisms and outcomes highlighted by family carers.

**Results:**

The themes and views of family caregivers resonate with those of health care professionals. Their overlapping anxieties related to business-driven care homes, uncertainty of families when making EOL decisions and the importance of symptom management referring to contexts, mechanisms and outcomes, respectively. Contexts specific to family carers were ad hoc information about services, dementia progression and access to funding. Not all family carers identified dementia as terminal, but many recognised the importance of continuity of care and knowing the wishes of the person with dementia. New mechanisms included specific resources for improving EOL care and barriers to discussing and planning for future care. Family carers identified the importance of comfort, being present, the meeting of basic care needs and feeling the right decisions have been made as good outcomes of care.

**Conclusions:**

Family carers and health care professionals share similar concerns about the challenges to good EOL dementia care. Better understanding of the effects of dementia at the advanced stages would improve confidence in EOL care and reduce uncertainty in decision making for family carers and health care professionals.

## Background

There is a global increase in the number of people living with dementia [1] and it will soon become a leading cause of death [2]. An increasing number of people with dementia are dying in long-term care settings in the United Kingdom (UK) [3] and in other countries such as Belgium, Canada and New Zealand [4]. Advanced dementia is characterised by dependency in all activities of daily living, inability to communicate needs and often multiple co-morbidities such as diabetes and hypertension [5]. The European Association of Palliative Care (EAPC) White Paper [6] provides a comprehensive definition of optimal palliative care for older people with dementia centred on an 11-domain framework to provide guidance for clinical practice, policy and research. Palliative dementia care should be continuous, proactive person-centred care with timely recognition of the dying phase whilst providing comfort, psychosocial and spiritual support and avoiding unnecessary burdensome treatments [6]. However, people living with advanced dementia do not routinely receive good care at the end of life (EOL) [7, 8].

The current organisation of the health and social care system in the UK may contribute to the inequities of care. In the UK, care for people living with dementia is provided by a combination of public and private health and social care systems. Health and social care funding in the UK is managed at a local level via Clinical Commissioning Groups (CCGs). Limited National Health Service (NHS) resources and funding means continuing healthcare is not readily available for all people living with dementia. Eligibility for continuing healthcare is based on needs which takes into consideration the complexity, intensity and unpredictability of needs (for example, a person living with advanced dementia who is uncommunicative and bedbound may not be eligible for continuing healthcare as their needs may not be deemed to be severe or a risk to their health). Currently those who are deemed ineligible for continuing healthcare may be eligible for NHS-funded nursing care but this is a means-tested scheme. Thus people living with dementia who have to move to a care home but who have savings over £23,250 and or own their own property would not be eligible and would have to cover the cost of their care provided in a care home. It has been suggested that the system is not designed to support people living with advanced dementia and their families [9].

Recent guidance from the National Institute for Health and Care Excellence (NICE) recommends that palliative care should be provided for people living with dementia from diagnosis while taking needs and the uncertainty of the disease trajectory into consideration [10]. General Practitioners (GPs) commonly provide primary care to people with advanced dementia residing in care homes and their own homes. Some care homes are supported by specialist palliative care services based within the health care system whilst others must rely on the charitable sector such as outreach teams from hospices. Similarly, specialist nurses (i.e., Admiral Nurses) provide support for people with dementia and their families throughout the disease progression and into bereavement for families. However, this service is provided by a charity and therefore access across the UK is variable [11].

Despite the increased need for optimal EOL care in dementia, there remains gaps in quality and consistency in long-term care settings, where provision is often uncoordinated and reactive [12–15] and many residents continue to experience unnecessary and burdensome interventions [16]. Similarly, a recent review of palliative dementia care interventions delivered at home has highlighted striking gaps in the evidence [17]. For example, although people with dementia experience burdensome transitions near the end-of-life with on average of two admissions in the last year of life [18], only one study explored the effect of a palliative care intervention on reducing burdensome and potentially unnecessary treatments at the EOL for those residing at home [17]. These findings highlight that although keeping people with dementia at home for longer is a key government goal [19] we know very little about how we can achieve this especially for those at the advanced stages of dementia.

Family carers provide most of the care and support to people with dementia [20]. Care can include 24-h supervision and addressing basic physical needs if the person with dementia is living in their own home to providing emotional and financial support for someone who resides in a care home. Compared with care provided to older ageing adults, family carers provide an additional 41.5 h of care per week to those with severe cognitive impairment [21]. Although caring can be a positive experience including an enhanced relationship with the care recipient, and a sense of accomplishment [22], high levels of depression and anxiety among such family carers are common [23]. The EAPC White paper makes a number of recommendations for addressing family carer needs. Family carers should be supported throughout the disease trajectory and into bereavement, and included in decision-making even if the person with dementia resides in long-term care settings. Finally, there should be continuous education about disease progression whilst assessing family carer understanding of the information [6].

Family carers are dissatisfied about information-giving about the dementia trajectory and support during the dying process and bereavement [24–26]. Bereaved family carers value good relationships with healthcare providers, and desire better information about the progression of dementia and a sense of control in EOL care [23, 27, 28]. There is a need to prioritise person-centred and compassionate care [26, 29]. Perhaps unsurprisingly, burdensome and sometimes futile interventions provoke family carer dissatisfaction [30].

In this paper, we present findings from qualitative interviews with family carers providing care for someone with advanced dementia living either in long-term care settings or in their own home. As in our study of healthcare professionals’ (HCPs’) perspectives on EOL care [31], we use a realist approach [32] to examine the contextual factors and processes affecting outcomes which are considered against EAPC standards [6]. A realist investigation explores the processes currently in place within the health and social care environment and seeks to explain what works for whom, in what circumstances and why [33]. This approach is useful for developing hypotheses using the context-mechanism-outcome configurations (CMOc) to present the underlying causal processes and their outcomes [33]. Consistent with a realist approach, our team developed an initial programme theory based on our current clinical knowledge and the literature [34]. This theory stated:

*EOL care for people with end stage dementia could be improved by increasing the depth of understanding of symptoms and unmet health and social care needs, allowing prompt recognition and appropriate confident management by professionals working in consultation with family carers. This understanding should be underpinned by improved awareness amongst all groups of the natural history of dementia and the significance of the end stage* [31].

We used this theory to underpin the topics discussed in our interviews with family carers. Although realist methodology has been used to evaluate interventions [35], the CMO can be used to disentangle the complexity of providing care to people with advanced dementia and define the contextual factors and processes (mechanisms) which can result in good and poor care outcomes. This is particularly important for informing interventions with strong underlying theory designed to improve EOL care for people living with advanced dementia.

### Aims

To examine:

1. the experiences of family carers of people with advanced dementia, their awareness and understanding of the contextual factors of care, and the mechanisms that are generated and considered to improve or hinder EOL care as an outcome.
2. the similarities and differences between the contexts, mechanisms and outcomes identified by the HCPs [31] and family carers.

## Methods

### Ethical review

Ethical approval was granted by the London – Bentham Research Ethics Committee (Reference: 12/LO/0346).

### Recruitment and description of participants

We recruited adults providing care for a family member with advanced dementia living in London, UK (severity of dementia was categorised as people who were 6e and above on the Functional Assessment Staging Scale (FAST) [36]). Suitable family carers were identified by research staff recruiting residents from 13 care homes (all of which were for-profit organisations) in London to a parallel cohort study within the COMPASSION programme [37, 38] and through the research team’s knowledge of other family carers regularly visiting the care home. Family carers participating in the cohort study [38] were provided with an information sheet about the current study. Family carers not in the cohort study were approached by the care home manager or sent a letter of invitation and an information sheet by post. If interested, the researcher arranged a convenient time and place for the interview. Family carers of people with advanced dementia residing at home were recruited through primary care using a similar approach.

A total of 14 family carers were recruited, of which seven were also taking part in the cohort study and caring for someone with dementia residing in a care home and six who were also caring for someone with dementia residing in care home but they were not participating in the cohort study. One family carer who was also not taking part in the cohort study was recruited through general practice as they were caring for someone living with advanced dementia residing at home. Written consent was obtained. Interviews took place between January 2013 and September 2013 and lasted approximately 1 h.

### Data collection

Our topic guide was developed from findings of a rapid literature review, workshops with family carers, people with early dementia and health and social care professionals, and interim analysis of the cohort study. The guide permitted flexibility to ensure sensitivity to participants’ self-expression in accounts of their own and to enable a discussion between the interviewer and participant [32].

### Data analysis

Interviews were audio-recorded, transcribed and entered onto a qualitative software programme (Atlas-ti) for coding. For accuracy, transcripts were checked against audio recordings. To achieve familiarisation with the data, a researcher (JH) read each transcript several times prior to coding and analysis. She then undertook the first step of thematic analysis [39] and applied codes to units of text. She used an iterative process of coding the data to ensure that each unit of text was assigned to the most relevant code(s) as new categories were developed. Three members of the team (JH, KM & NK) reviewed the codes and eliminated any codes which were not related to EOL care. Two members of the team (KM & NK) each reviewed half of the units of text and their assigned codes relating to EOL care. To achieve the first aim of this study, we reviewed the codes together with the units of text assigned to them and categorised them as contexts, mechanisms and outcomes. We completed the second aim by comparing and contrasting the themes developed from interviews with family carers to context, mechanism and outcome themes developed during interviews with HCPs from our earlier work [31]. We identified which of the HCPs CMO were evident in the family carer codes and where there were discrepancies. New CMO in the family carer data were added to the existing framework and relevant quotes selected for inclusion in this paper. Finally, we identified common links between CMOs and present them as CMOc. The CMOs represent how the relationship between contexts and mechanisms lead to good or poor EOL care outcomes for people with advanced dementia based on the family carer experience. Minor grammatical changes have been made to some quotes for ease of reading.

## Results

We recruited 14 family carers (7 daughters, 5 sons and 2 wives). Most (*N* = 13) cared for a family member residing in a care home whilst one was caring for a person with advanced dementia living at home. Length of residence in care homes was between 1-6 years.

We begin by presenting the key themes and sub-themes that set the context for good EOL care. This is followed by themes that represent mechanisms and processes that were generated and considered as improving or hindering EOL care outcomes. The third section examines themes relating to the outcome; what constitutes good EOL care in dementia. For each of these sections we compare themes and sub-themes with those identified by the parallel study with HCPs [31]. We provide greater detail on the new themes identified through the family carer interviews that were not evident amongst HCPs. Finally, we draw out connections between contexts, mechanisms and outcomes as CMO configurations (CMOc).

### Context of care

We found eight themes relating to the context of EOL dementia care: business driven care homes; a complex network of health and social care providers; societal and family attitudes towards care home staff; staff training, experience and reflective processes; governance and regulation of care homes; complexities of providing care in advanced dementia; advance care planning and; staff personality/characteristics. These themes were all also identified from the HCPs interviews (See Table 1). However, family carer data revealed two additional themes: information needs of family carers and cost of services.

**Table 1:** Context themes for healthcare professionals (HCP [31]) and family carers

Theme	Sub-theme	Reported by:	
		HCP	Family carers
Business driven care homes	Profit prioritised over care quality	✓	
	Lower staff salaries and lowly skilled care staff	✓	✓
	Minimal staffing levels	✓	✓
	Poor staff conditions	✓	✓
	Increasing turnover of staff, ***lack of continuity***	✓	✓
	Negative image of care homes and low prestige working in care homes	✓	✓
	Demanding workloads	✓	✓
	Staff have limited time	✓	✓
Complex network of health and social care providers	Multiple agencies to make referrals to and communicate with	✓	
	No option to make direct referrals from care home	✓	
	Long waiting times for some services/***care home admission***	✓	✓
	External HCPs who are proactive and helpful in providing care to people with advanced dementia/***access to external services***	✓	✓
Societal and family attitudes towards care home staff	Negative perception of care homes	✓	✓
	Recognition that care home staff work hard	✓	✓
	Lack confidence in care home staff	✓	✓
Staff training, experience and reflective processes	Lack of training/experience in dementia care ***(in care homes, hospital and amongst GPs)***	✓	✓
	Post-death reflections	✓	
	Beneficial to prepare staff for EOL care and to provide exposure to EOL care	✓	
Governance and regulation of care homes	Highly regulated	✓	✓
	Excessive documentation and scrutiny	✓	✓
Complexities of providing care in advanced dementia	Long trajectory and unpredictable prognosis	✓	
	Challenging to manage symptoms due to the communication difficulties	✓	✓
	Difficult to understanding the relationship with palliative care	✓	
	Palliative care services not equipped to manage behavioural symptoms of dementia	✓	
	Need for continuity of care and gradual changes		✓
	Difficult decisions regarding quality of life and prolonging life; can no longer have EOL conversations with person who has dementia		✓
	Stigma associated with dementia impacts on care		✓
	Dementia not considered terminal		✓
Advance care planning	Proactive Advance Care Planning	✓	✓
	Importance of involving GP and family in these discussions	✓	✓
Staff personality/ characteristics	Compassion	✓	✓
	Motivation/***making an effort***	✓	✓
	Initiative	✓	
	Finding the job rewarding	✓	
Information needs of family carers	Lack of formal structure to provide information to support family carers		✓
	No single point of contact for information regarding resident’s health		✓
	Family rely on information from the Internet and brochures, Admiral nurses helpful		✓
	Family carers feel unprepared and ill-informed – don’t know what or who to ask		✓
Cost of services	Some family carers can pay for better services and some experience financial burden to pay for services		✓
	Inadequate funding for continuing care		✓

*Note.* Bold, italicised text indicates additional detail to sub-themes added after analysis of family carer interviews

#### Contextual themes identified by both HCPs and family carers

Similar to HCPs, family carers also identified the business-driven nature of care homes, the level of scrutiny and governance of the care home sector and societal and family attitudes to care home staff. Both highlighted poor staff salaries, low staff-patient ratios, demanding workloads and low skill base, as exemplified by this quote from a family carer:

*“I always tell the manager downstairs, when you have your meetings, don’t forget to praise them. It’s nice to have praise because they are doing a job that I can’t do and it’s a difficult job… It’s a difficult job and I think that if they were better trained, it’s not their fault. It’s the ones higher up the ladder, they are not doing it properly. They need supervision, they need to be trained properly to give the people the best you can. And it all boils down to money I’m afraid… They need supervisors and the supervisors must know the job, not only how to treat the patients and their families but also the staff, because it is the staff who are the front-line workers.” (ID: 5)*

HCPs distinguished between care homes and health services such as GPs and hospitals; care homes were considered as delivering a lower quality of care. Family carers, felt that care home staff were better equipped to provide EOL dementia care due to greater experience and were concerned about hospital staff and GPs’ skill in meeting patients’ needs:

*“Although I think the hospital say they understand dementia, they don't. The nurses on the ward do not understand dementia, and I think actually those nurses on the ward should be taken off, especially ones on geriatric wards… into a care home, a pure dementia unit, for a month and be made to work with it, because it's the only way you start to get your head around it. It's so alien to get into the head.” (ID: 12)*

Family carers were distressed about long waiting times for services and care home admission, noting HCP variation in supporting and promoting access to services such as mental health. Family carers described the importance of staff personality and characteristics (e.g. compassionate, motivated and made an extra effort):

*“Yes, it was warm, it was compassionate, like I said, I’ve seen what happens when two or three other people have gone through a long decline. People pop into their rooms just to hold their hands. That’s not something that anybody pays them to do or asks them to do. They do it because they know that people are on their way out, which is…when you see that you feel rather reassured.” (ID: 9)*

The contextual complexities of advanced dementia care provision and advance care planning were expressed differently in family carer and HCP interviews. HCPs focused on the suitability of palliative care services and the unpredictable nature of dementia. Some family carers did not recognise dementia as a terminal condition. Family carers also described the need for continuity of care, the difficult decisions regarding balancing quality of life with prolonging life, the inability to discuss EOL care with the person with advanced dementia as well as the stigma of dementia impacting on the quality and range of services available.

*“It’s not about me. It’s about how he’s feeling. When he doesn’t eat how does it affect him? I can’t feel for him. Force feeding them is wrong, because you are just prolonging the time and the more you feed, they get a bit of strength out of it I suppose and it’s just making his time here longer when it should be shortening. I don’t want him to go and that is selfishness on my part, yet I know it’s best for him that he wouldn’t have to go through all that.” (ID: 5)*

#### Context themes identified by family carers only

Family carers also reported limited information-giving and high service costs but these were not raised by HCPs. While numerous helpful information sources were identified, such as information from the Internet, brochures and Admiral Nurses (specialist dementia care support), family carers were dissatisfied with the timing and access to this information, often ad hoc or not at all.

*“Through the hospital, they said, ‘Look, Royal National Institute for the Blind have some really brilliant stuff.’ So I went to the website and bought these things. But so much was by chance, so much was what you Google and by mistake found this, found that. And that’s not the way it should be.” (ID: 3)*

Lack of information commonly left family carers unsure about fundamental aspects of dementia and not knowing whom to contact:

*“I think I would have liked more information about her illness … because they talk about people having Alzheimer’s, and they talk about people having dementia, and I'm not sure the difference between Alzheimer’s and dementia, are they two separate illnesses, or is dementia just a different form of Alzheimer’s? I'm still not absolutely sure about that.” (ID: 7)*

Costs of services and availability of NHS continuing healthcare were contextual barriers to accessing services. This funding enables those who are eligible to receive care outside of hospital that is arranged and funded by the NHS. Eligibility is defined as having a primary health need and is assessed by a multidisciplinary healthcare team. Severity and complexity of need is taken into consideration. Some family carers perceived that better services such as one-to-one care were available but conditional on additional payments. Financial pressures forced families to decide between budgeting for high level care for a family member or their own personal financial security:

*“We have the house and can afford to go for the home we wanted. ….. I mean my dad would have been upset that we...well, I don't think he would have been upset that we sold the house, but his thing, 'No, that house is always for you three girls,' and I always think he's...he always planned on it being our security for our old age. Well, that isn't particularly going to happen.” (ID: 12)*

### Mechanisms generating outcomes

Our HCP data indicated four themes relating to mechanisms which included: level of HCPs confidence; resources for improving EOL care and supporting families; family uncertainty about EOL care and; Clinical Commissioning Groups (CCGs) uncertainty about whether dementia specific palliative care is required. We also identified these themes in interviews with family carers, with two additional themes; family carers’ confidence in care staff and family carers’ determination in accessing NHS continuing healthcare funding (see Table 2).

**Table 2:** Mechanism themes for healthcare professionals (HCP [31]) and family carers

Theme	Sub-theme	Reported by:	
		HCP	Family carers
Level of HCPs confidence	Confidence/uncertainty about best approach to EOL care	✓	
	Fear of litigation	✓	
	Fear of death (avoidance)/Accepting (comfortable with dying/death)	✓	
Family carers’ confidence in care staff	Family lacking confidence in care quality leading to supplementing and monitoring care		✓
Family uncertainty about EOL care	Confusion/uncertainty regarding EOL care decisions, ***particularly around food and eating***	✓	✓
	Family avoiding discussions regarding EOL	✓	✓
	Family carers not recognising the need/importance of having these conversations or who to go to		✓
	Family carers lacking information to inform decisions and unaware that ACPs can be altered; difficulty evaluating care quality		✓
Resources for improving EOL care and supporting families	Admiral nurses	✓	✓
	Post-death reflections	✓	
	Building relationships between people with dementia, family carers, care staff and healthcare professionals		✓
	Observation and experiential learning as more effective (particularly where staff have limited writing skills)		✓
	Advance Care Planning, in particular DNAR as a resource for comfort care/good death		✓
	Gold Standards Framework as a resource to provide good care but variable use across care homes		✓
Family carers’ determination in accessing NHS continuing healthcare funding	Limited resources and a poor understanding of the complex needs of those with advanced dementia appear to restrict access to continuing care funds		✓
CCGs uncertainty about whether dementia specific palliative care is required	Uncertainty as to whether specific dementia palliative care services are necessary	✓	

*Note.* Bold, italicised text indicates additional detail to sub-theme added after analysis of family carer interviews

#### Mechanisms identified by HCPs and family carers

Most of the core mechanisms were identified by both HCPs and family carers. The first three themes listed in Table 2 are all variations of a theme that relates to lack of confidence and uncertainty in the context of dementia and EOL care. We have reported them separately as they operate in different ways with different effects. HCPs attributed their own lack of confidence in EOL dementia care to the nature of advanced dementia and the difficulties of determining the best approach to care. Families also had difficulties in determining the best approach to care but this was attributed to their confusion and lack of information and knowledge about dementia, avoiding discussions about EOL care or not recognising the importance of having these conversations. They hinted that HCPs were not being honest and open with them about prognosis.

*“I don’t think that as a society our end of life care is very good. We’re not good at explaining to relatives what's happening. Again it's looking forward. Why didn’t someone say, ‘Your father’s not very well, your father is really quite ill,’ sort of thing. And you start to learn at a point that they're telling you he's going to die in the next…but you don’t actually know, the next day, week, you just, and they know perfectly well.” (ID: 13)*

Family carers also identified additional mechanisms or resources for improving EOL care that were not identified by HCPs. These included the Gold Standards Framework (GSF; national UK framework of staff training and education for improving EOL care for people with life-limiting conditions, including those residing in care homes), Do Not Attempt Resuscitation (DNAR) orders and the importance of building relationships between people with dementia, family carers, care staff and HCPs.

*“I had signed a 'Do not resuscitate' form... I want her to pass away there [in the care home]. The way I feel, if that is her home now, she feels safe with those people, I see the way she responds to the carers, she's never stressed… we signed the 'Do not resuscitate' form and … if and when... when something happens, we just want her there… a neighbour of mine, her mum was in there, and she wouldn't sign the 'Do not resuscitate' form and her mother passed away in there. It was absolutely horrendous because the ambulance came… they tried to resuscitate her... the whole thing was awful. So her lasting memory of her mother is lying there with tubes and lying on a floor where they'd been bumping up and down on their chest. Is that how you want to remember your mum?” (ID: 12)*

Family carers highlighted the importance of training staff in dementia care, emphasising the need for observational and experiential learning experiences for staff (particularly where staff had limited written English skills):

*The other day, they [care home staff] were all going for training here... I think that it would make it much easier for them to see actually somebody do it than have to write down… They need to be shown as well as the paperwork. I think in all walks of life, you need to be shown what to do. (ID: 5)*

#### Mechanisms identified by family carers only

Two key mechanisms were noted in the family carer interviews: family carers’ confidence in care staff and family carers’ determination in accessing NHS continuing healthcare funding. Some family carers lacked confidence in the ability of care home staff and HCPs to provide suitable care for their relative, attributed to poor skills and understanding of dementia and a lack of time. Family carers who lacked confidence in care quality tended to try to supplement and monitor care through supporting their relative with meals and spending considerable amounts of time with their relative either in hospital or the care home.

*“I mean, I'll be honest, the reason we go every day is to keep an eye on her, and I think just don't take your eye off the ball, yeah? Never take your eye off the ball.” (ID: 12)*

Family carers also discussed their determination in accessing NHS continuing healthcare funding. Family carers felt that dementia was not always fully considered as a healthcare need and that gaining access to this limited resource was challenging and required considerable determination:

*“I'm hoping with the care package… the NHS Continuing Care Package that I can step back a little bit... I wouldn’t have got that without consultation from a solicitor because I was told no by the Social Services, I was told no by the NHS, and I wouldn’t take no for an answer because I knew that my mother’s complex needs deserved it. But it was a huge battle, a huge battle to get it”. (ID: 11)*

### Outcomes defining good EOL dementia care

This section examines the themes that reflected how family carers and HCPs defined the outcomes of good EOL care in dementia. We also provide examples where the expectations of family carers were not met. Table 3 provides the key outcome themes for HCPs and family carers. HCPs identified four themes relating to outcomes including psychosocial and spiritual care; addressing physical needs; supporting and developing relationship with family carers and; continuity, integration and multidisciplinary care. Family carers identified almost all the themes and sub-themes referred to by HCPs along with one additional theme labelled as ‘EOL care provided at home/homelike environment’ and several additional sub-themes.

**Table 3:** Outcome themes for healthcare professionals (HCP [31]) and family carers

Theme	Sub-theme	Reported by:	
		HCP	Family carers
Psychosocial and spiritual care	Beyond meeting basic physical needs	✓	
	Person-centred approach	✓	✓
	Spending time with residents	✓	✓
	Treated with dignity and respect	✓	✓
	Being seen by a religious figure e.g. priest	✓	✓
	Resident is not alone, is engaged and has comforting physical contact		✓
	Is comfortable, warm, content and feels secure; death is quick and peaceful		✓
Addressing physical needs	Symptom management (particularly for pain)	✓	✓
	Reducing burdensome interventions, hospitalisation and resuscitation	✓	✓
	Basic care needs are understood and met; e.g., clean, not smelling, hearing aids in place		✓
	Has improvements in health, increased life expectancy		✓
	Good food and support for adequate nutrition and hydration		✓
	Harm is minimised (e.g., falls, bruises)		✓
Supporting and developing relationship with family carers	Collaboration between family and care home staff	✓	✓
	CH staff getting to know the family and obtaining trust	✓	✓
	CH staff helping ***(supporting)*** family carers to prepare for their relative’s death and discussing grief	✓	✓
	CH staff providing support	✓	✓
	Family carers feeling prepared with plans in place, involved and informed; not making decisions under pressure		✓
	Family carers feeling that the right decisions have been made		✓
Continuity, integration and multidisciplinary care	Good working relationships across services	✓	✓
	Regular staff who get to know individual needs of residents	✓	✓
EOL care provided at home/homelike environment	Home or homelike environment makes it more familiar, relaxed and safe and therefore more comfortable for person with dementia and family		✓
	Care homes more homely and preferable to hospital		✓

*Note.* Bold, italicised text indicates additional detail to sub-themes added after analysis of family carer interviews

#### Outcomes identified by HCPs and family carers

Psychosocial and spiritual care were important amongst family carers and HCPs and included providing person-centred care, spending time with the person with dementia, treating them with dignity and respect and offering religious services. Symptom management (particularly pain management) and reducing burdensome interventions, hospitalisations and resuscitation were seen as good outcomes in EOL for both HCPs and family carers:

*“I won’t have him resuscitated if it happens. He’s got to go the natural way. I don’t want to take him to hospital and they do all these things to him and I think what for? If he was going to get better, you would, but it is not going to do him any good. Well, I don’t know what happens to him when they start pulling him around, how he feels, but it’s not going to make him better. It just prolongs the agony” (ID: 5)*

Collaboration between families, HCPs and care staff were reported as good outcomes by both HCPs and family carers and involved care home staff getting to know and obtain trust from the family and supporting family carers to prepare for their relative’s death. Continuity, integration of services and multidisciplinary care were also important facets of collaborative care and relationship building identified by HCPs and family carers.

#### Outcomes identified by family carers only

Family carers provided a larger range of specific care elements that reflected good EOL care. Sub-themes relating to psychosocial and spiritual care that were only reported by family carers included the person not being left alone and isolated, having opportunities for social engagement and physical contact as well as being comfortable, content and that death is quick and peaceful:

*“Most of all I would want her to have peaceful death. I don’t want her to suffer any more than she is suffering now.” (ID: 9)*

Four sub-themes under the theme of addressing physical needs were only reported by family including understanding and addressing basic care needs, prolonging life and identifying when it was appropriate to provide treatment:

*“No, she has never been on death’s door. She’s been in a lot of pain and agony through urinary tract infections and stuff like that, and they’ve treated those, but I don't think the doctors felt that was going to kill her so, therefore, I don't think they felt they could allow that condition to take her to be an angel.” (ID: 3)*

Other sub-themes relating to physical care needs included minimising harms and providing adequate nutrition and hydration. However, there were numerous examples of physical needs and safety not being met with reports of abuse, bruising, pressure sores, falls, dehydration, infections, unmanaged pain and isolation suggesting that services were failing to minimise harm and neglect.

*[During 3 weeks residential respite while carer was hospitalised] “I think one of the carers hit him or... He was all black and blue… Yeah. He was beaten... I was supposed to have a couple of days here to convalesce on my own, but I couldn't. I had to just go and get him out, and I did. So I was dressing him and I looked, he was all bruised. When I asked what happened, he said, 'Oh, I got beaten,' and I said, 'What?' He said, 'Yeah, they weren't very nice to me.'” (ID: 10)*

Additional sub-themes under the theme of supporting relationships included having plans in place so that decisions did not have to be made under pressure, being kept involved and informed, and feeling that the right decisions were made.

*“She wouldn’t survive anyway I don’t think, having resuscitation, and I don’t think it would improve the quality of her life. I mean, it [signing a DNAR] was a hard thing to do because again, you feel as though you're failing her, you feel as though you're giving up on her. But the nurse I spoke to here said, ‘It's not set in stone, they'll always phone you to let you know what's happening.’ But we've decided that when it does come that just…it's in the best interests for mum as well, make it as peaceful as possible for her.” (ID: 7)*

One theme relating to good EOL care that was raised by family carers but not HCPs was the importance of care being provided either at home, or in a homelike environment. For most of the family carers, their relative was residing in a care home and having a homelike environment in the care home helped it become an alternative to home and a preference for place for death over hospital:

*“I think they’re really, really good [in care home]. They’re friendly to us when we come in. I think it’s got a very, very homely atmosphere, which the other place didn’t have at all. I know this is smaller...it wasn’t at all homely. I think the things they’ve got, the pictures on the wall, the decor, it’s light and airy, and it’s stimulating’. (ID: 4)*

Only one family carer in our study was still providing care for their relative at home and they saw this as crucial for their relative remaining alive and for a comfortable death:

*“Every single carer that's ever looked after mum has always said to me, she wouldn’t last 2 weeks in a care home because she is aware, she knows that she's in familiar surroundings. She knows that I am hers, she says, ‘Mine,’ she doesn’t know my name, I don’t care whether she knows my name or not, it's irrelevant. I don’t care if she knows whether I am hers or not, I'm there for her and that's all that matters… I want mum to die at home in familiar surroundings. I will do my very utmost to afford her that facility, if you like because I saw her suffering so much in hospital, so much, and she couldn’t express it”. (ID: 11)*

### Context-mechanism-outcome (CMOc) configurations and realist explanations

Based on the contexts (C), mechanisms (M) and outcomes (O) described above we suggest two mid-level theories, or CMO configurations (CMOc), which set out how the relationship between contexts and mechanisms lead to good or poor EOL care outcomes for people with advanced dementia based on the family carer experience.

#### CMOc 1

Continuity of care staff, staff who understood dementia, had adequate time to do their job (C) were contextual factors that supported establishing good relationships between staff, family carers and people with dementia (O). These contextual factors also meant that staff could get to know the resident better and therefore became better at understanding and addressing individual needs (O). Contexts that supported relationship building helped family carers feel confident in the care (M) and created a cycle of further relationship building. Family carers also viewed their own regular visiting to the care home as part of the relationship building and getting to know the care home staff.

*“I have to say for the family members who go regularly to visit people at my mother’s care home, there are regular conversations, on-going conversations with nurses about feeding, about medication, about whatever, we are involved. So there’s an expectation, we’ve been told that we would obviously be talking to the GP if something happened.” (ID: 9)*

However, when these contextual factors were not present and care home staff were overstretched, lacking continuity and were perceived to be there for the pay only (C), relationships and person-centred care was not fostered (O), family lost confidence in the care and become vigilant in monitoring and supplementing care (M) and relationships broke down (O). While regular visiting was viewed as a method of fostering relationships with staff, it was also a method for families to monitor care due to lack of confidence.

*“Because I was there on a daily basis and was very sensitive to changes in mum. If she was ill, if she was constipated she would manifest this because she would start to squirm a lot and bend forward, and sometimes they hadn't always checked how many bowel movements she had had. I used to go and get the book out and flick through and look, 'Oh, bowel type 5, bowel type 4,' or whatever. That's because I was there all the time. Now it might be very different from somebody who, for example, is visiting on a weekly basis, a monthly basis, or sadly, never visits at all.” (ID: 1)*

#### CMOc 2

**Figure 1:**
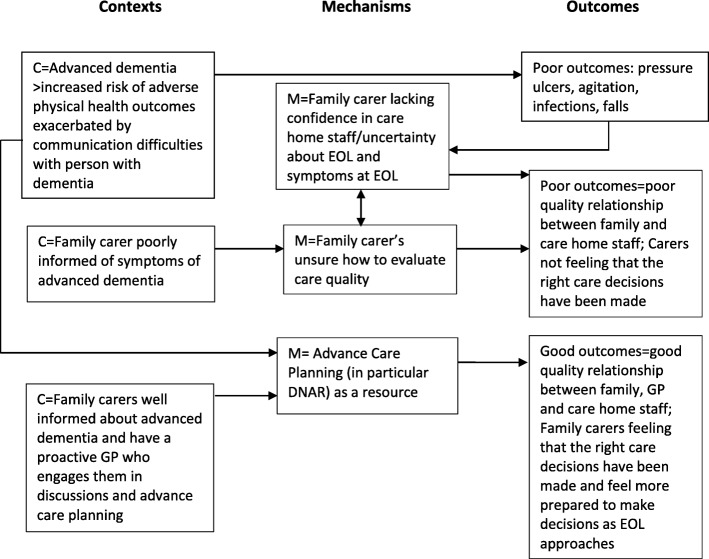
Illustration of links in CMOc 2

The second CMOc relates more directly to the complexities associated with dementia care during the advanced stages of disease and the importance of information provision and helping family carers plan for EOL care. People with advanced dementia have complex needs and have difficulty communicating these needs (C). This puts them at greater risk of poor outcomes such as pressure ulcers (O). Poor outcomes may be prevented with good quality care, but may also be difficult to prevent even when care quality is high. Family carers are poorly informed or unaware about common symptoms associated with advanced dementia (C) and may have difficulties in how to evaluate care quality (M). Family carers have low confidence in care home staff (M) which is either due to these poor outcomes occurring or is reinforced by these symptoms occurring for which family carers may not be prepared for. Their low confidence in care home staff leads to poorer quality relationships with staff (O) creating a complex set of factors impacting on one another as illustrated in Fig. 1.

Family carers expectations that their relative with advanced dementia may experience health improvements may be unrealistic.

*“Well, at least she is still alive because like I said a while back her condition was so bad like she wouldn’t make it. So, you know there’s a lot of improvement so I’m very satisfied.” (ID: 2)*

*“Recently they have actually self-reported themselves to the Care Quality Commission for mother’s bedsores and they involved Social Services, they asked me my views. I said, ‘Well, I can't really express views.’ If I hadn't been told mother had bedsores I wouldn't know.” (ID: 3)*

In contrast to this poor set of outcomes influenced by poor information provision and lack of planning, we have also incorporated in CMOc 2 (see Fig. 1) the scope for more positive experiences of EOL care for family carers. A more positive outcome occurs when family carers do have access to good information about advanced dementia (C) and a GP who promotes and supports advance care planning (C). This leads to family carers feeling more involved in decision making (M) and more likely to tackle difficult EOL care discussions that tend to be delayed and avoided:

*“I don’t know, it's a bit of a difficult one as I said, because everyone’s different, there are people that are quite able to have these discussions within their family, and talk about all different things. But there are other families that are more…what is the word? Private. They're more private about their emotions, about different things as well, and they're not able to open up and talk about it”. (ID: 1)*

Supporting family carers to have these difficult discussions also helped foster good relationships between care home staff, GPs and family carers (O). This also helped family carers feel more prepared for EOL and for making urgent decisions as EOL approaches and helps them feel that the right care decisions have been make (O).

*“They [care home staff] know that should anything happen that in the 24 h, the nurses will come and make sure that he’s pain free and all that sort of thing. And I’m quite happy with that because I know that they’ll do that. They’ve told me that they’ll do that so he’ll be pain free and comfortable”. (ID: 5)*

*“But actually, shouldn’t that conversation [about end of life care] happen sort of at my stage almost now and I haven’t had, you know, and I have signed a piece of paper while standing in the nurses cubicle, you know, a DNR thing and I haven’t had a conversation with the GP… maybe that is something that should actually happen as an outcome. A piece of paper where a conversation has happened about end of life care”. (ID: 9)*

## Discussion

To our knowledge, this is the first study of its kind to explore and compare family carers and HCPs views of EOL dementia care and how contexts and mechanisms lead to good or poor EOL care outcomes. We used a realist approach to highlight similarities with knowledge derived from analyses of data from HCPs and add some important new findings. Based on these comprehensive analyses, we make recommendations on how services may enhance positive contexts and mechanisms to facilitate good EOL dementia care.

### Similarities of findings between HCPs and family carers

Most contextual themes and sub-themes were shared by HCPs and family carers. Both groups recognised the business-driven nature of care homes, the complexity of the health care system, the impact of staff training and attitudes and the complexities of providing care in advanced dementia as challenging contexts to providing good EOL dementia care. While both groups identified staff attitudes, family carers tended to put a greater emphasis on the extent to which staff made an extra effort to provide good care, indicating that some were just there for the pay while others had a genuine concern for their relative and for providing good quality care. This perceived effort was in the context of staff who had limited time and skills. It must be highlighted here that the care homes in both studies were for-profit organisations. Previous research has shown that care quality can differ between for-profit and not-for-profit organisations with a number of outcomes including the number and quality of staffing with not-for-profit care homes employing more or higher quality staff [40].

Family carers also identified most of the outcomes identified by HCPs reflecting good EOL care including psychosocial and spiritual care, addressing physical care needs, developing relationships and providing continuous, integrated and multidisciplinary care. There were similarities in the mechanisms for good EOL care but they tended to be framed differently. These findings are supported by previous research which have highlighted that both family carers and HCPs report that there should be better awareness and observance of the person with dementia’s wishes at the end of life [41]. This is particularly important for HCPs who may recommend invasive and burdensome interventions at the end-of-life against the wishes of the person with dementia [41].

### Additional themes identified by family carers

The contextual factor reported solely by family carers was that the systems for providing information to family carers about service costs and access, dementia progression and its effects in the advanced stages were poorly organised and available on an ad hoc basis. Family carers felt that good information was available, but locating information in a timely manner was often not achieved. Provision of information about the progressive course of dementia is an important element of EOL care in the EAPC White paper [6]. This lack of information about disease progression has also been identified as a gap in previous studies in a number of countries [23–26]. Family carers also observed that to receive good quality services they needed to pay more for them, raising issues of equity in service delivery.

Mechanisms raised by family carers and by HCPs tended to relate to confidence in EOL care and uncertainty in decision making. HCPs found that the nature of dementia created uncertainty in EOL care, while family carers felt their own poor knowledge of advanced dementia combined with the poor knowledge and skills of care home staff and other HCPs meant that decision making was stressful with no clear process or expertise to guide care decisions. However, training and education of HCPs may not be sufficient to improve care. A recent realist review of interventions to improve care of people with dementia in hospitals suggest that senior members of staff should support care staff to implement their new knowledge and endorse good dementia care to improve staff confidence [42].

Our second CMOc illustrates how confidence can be influenced in a number of ways. If family carers are poorly informed about common symptoms at EOL, they may consider care to be poor because their relative is experiencing these symptoms. For example, the literature recommends comfort feeding in advanced dementia as a less invasive alternative to artificial feeding, however, the risk of aspiration is still present [43]. Many family carers in this study were concerned about burdensome interventions and the difficulties in decision making at EOL. Support with eating or artificial hydration and nutrition were difficult issues for family carers who were unsure whether their motivations were based on their own needs for their relative to continue living rather than the best interests of their relative with dementia. These difficult decisions created major dilemmas for some family carers while some indicated that their relative living longer than expected was an indicator of good care. These findings illustrate the difficulties that family carers have in how to evaluate care quality.

There is a complex interplay between the confidence of family and staff, knowledge of and management of common symptoms of dementia and how family evaluate care quality. This can lead to the apparent contradictory position where family carers want to develop good relationships with care home staff while sometimes becoming hypervigilant in monitoring care being provided. This could lead to staff feeling mistrusted and resentful. Communication and conflict-resolution training for family carers and care home staff have been found to have benefits including reduced behavioural symptoms for residents, improved communication and reduced conflict between family and staff and reduced staff burnout [44].

Family carers also identified a number of resources for improving EOL care that were not identified from the HCP interviews. While both groups identified the resource of Admiral Nurses, only family carers reported the need for observational and experiential learning (including hospital staff undertaking placements in care homes to observe care of residents with dementia), advance care planning and DNAR and the Gold Standards Framework to support staff development. They also identified their own determination as being a requirement to access limited NHS continuing healthcare funding. They felt they needed to fight for this funding and that advanced dementia was not necessarily assessed as a condition requiring constant care and supervision by assessors, thus impacting on eligibility.

Family carers reported more specific themes relating to the outcome of good EOL care. They noted, in particular, the importance of EOL care being provided in a home or homelike environment [30]. This was related to increasing familiarity and comfort for the person with dementia. Feeling secure, warm, not being alone, death being quick and peaceful and basic care needs being met were also reported by family carers. As reported above, for some, good care meant that their relative would live longer while others were keen to avoid burdensome interventions or transfers to hospital.

Good EOL care outcomes for family carers not raised by HCPs also connected the importance of good relationships with healthcare providers so that care was planned in advance and that decisions did not have to be made in haste. If this occurred family carers would feel more assured that good care was provided and that they could feel reassured that the right decisions had been made. This possibly has important consequences for the family carer’s grieving process post death [23].

### Implications for research, policy and practice

Our work highlights the importance of relationships between HCPs, care home staff and family carers in EOL dementia care. Fostering good relationships between family, HCPs and care home staff requires a collaborative approach. Although our findings indicate that there are gaps in information provision from HCPs and care home staff to family carers, communication and information sharing between family carers and HCPs is a complex and dynamic relationship. It must be acknowledged that family carers play an important role in how information and knowledge is shared [45]. However, where family require regular feedback about the health and wellbeing of their relative they would benefit from a single key contact who knows their relative well. This may help address uncertainty and gaps in information about dementia, enable honest discussions about the trajectory of dementia early on and EOL care symptoms. If this contact could provide reassurance that they are aware of the changing needs of the person with dementia, the family would be likely to feel more reassured that their relative is being well cared for. A regular primary care physician who is integrated into this relationship might provide reassurance to family carers and support to staff about care decisions and may enable development of advance care plans that help to support care home staff in how to respond to specific symptoms. The provision of a single key contact and a regular primary care physician throughout the disease trajectory would encourage the person living with dementia and their family to think about their needs and wishes for the EOL earlier. In line with a human rights perspective [46], these discussions may ensure the needs and wishes of people living with dementia are heard and met in a timely manner.

In line with our work with HCPs, our findings from interviews with family carers confirmed that higher systemic changes need to be addressed to improve EOLC care outcomes for people living with advanced dementia. Changes to the process of assessing eligibility and allocating funding for care costs, in particular from the NHS continuing healthcare system, are required. Inequities in access to funding in the UK demonstrate the importance of stream lining this process to ensure people with complex needs are assessed in a systematic and transparent way.

Our data support and increase our understanding of our original realist programme theory. In particular we have gained insights into the importance of integrating the needs of both informal and professional carers in considering end of life care for people with advanced dementia. Training and support for informal carers was identified as a key component in our COMPASSION intervention [34, 47]. The COMPASSION manual is available for download (https://www.ucl.ac.uk/psychiatry/sites/psychiatry/files/the-compassion-intervention-manual.pdf).

### Strengths and limitations

We have been able to compare and contrast the findings from HCPs with the views of family carers to ensure we have a comprehensive picture of the CMOs in EOL dementia care. However, the analysis approach may have introduced bias in themes identified. While the initial coding of family carer interviews was undertaken independently of the HCP analysis, there was a risk that the CMOs identified in the HCP analysis were more likely to have been identified in the family carer analysis. However, new CMOs were identified and some of the HCP themes were not identified in the family carer analysis indicating that that there was some independence in the two analysis processes.

While the sample size was small, it was sufficient to identify CMOs to inform practice. We cannot claim that our findings are representative of all family carers of people with advanced dementia, including former carers, and some issues may not have been identified. In particular, we were able to recruit only one family carer of someone with advanced dementia living in their own home and our findings tend to focus on issues within the care home sector.

## Conclusions

This study adds to the existing literature by providing a comprehensive picture of the context and mechanisms influencing EOL care in dementia. During the advanced stages of dementia people have limited capacity and verbal communication and so open communication and trusting relationships between family, care staff and HCPs become paramount in providing a coordinated approach to EOL care. In addition to good relationships, increased provision of resources such as Admiral Nurses, staff training and advance care planning may help to reassure family carers that their relative will have a comfortable and peaceful death in a homelike environment.

## Data Availability

The dataset used and/or analysed during the current study are available from the corresponding author on reasonable request.

## Abbreviations

(HCPs): Health Care Professionals
CMOc: Context-Mechanism-Outcome configuration
DNAR: Do Not Attempt Resuscitate
EAPC: European Association for Palliative Care
GP: General Practitioner
NHS: National Health Service
NIHR: National Institute for Health Research
WHO: World Health Organisation

## References

[1] Alzheimer’s Disease International, World Alzheimer Report 2015: The Global Impact of Dementia. An analysis of prevalence, incidence, cost and trends, M. Prince, et al., Editors. 2015, Alzheimer's Disease International: London.

[2] Etkind S (2017). How many people will need palliative care in 2040? Past trends, future projections and implications for services. BMC Med.

[3] Sleeman KE (2014). Reversal of English trend towards hospital death in dementia: a population-based study of place of death and associated individual and regional factors, 2001-2010. BMC Neurol.

[4] Reyniers T (2015). International variation in place of death of older people who died from dementia in 14 European and non-European countries. J Am Med Dir Assoc.

[5] Poblador-Plou B (2014). Comorbidity of dementia: a cross-sectional study of primary care older patients. BMC Psychiatry.

[6] van der Steen JT (2014). White paper defining optimal palliative care in older people with dementia: a Delphi study and recommendations from the European Association for Palliative Care. Palliat Med.

[7] Sampson EL (2010). Palliative care for people with dementia. Br Med Bull.

[8] Sampson EL (2005). A systematic review of the scientific evidence for the efficacy of a palliative care approach in advanced dementia. Int Psychogeriatr.

[9] The All Party Parliamentary Group on Parkinson’s. Failing to care: NHS continuing care in England. London; 2013.

[10] National Institute for Health and Care Excellence. Dementia: assessment, management and support for people living with dementia and their carers 2018 [cited 2019 16th August]; Available from: https://www.nice.org.uk/guidance/ng97. 30011160

[11] Hope T (2010). Ethical issues and dementia: the Nuffield report. Clin Ethics.

[12] Davies N (2014). Barriers to the provision of high-quality palliative care for people with dementia in England: a qualitative study of professionals’ experiences. Health Soc Care Community.

[13] Gage H (2012). Integrated working between residential care homes and primary care: a survey of care homes in England. BMC Geriatr.

[14] Goddard C (2013). Providing end-of-life care in care homes for older people: a qualitative study of the views of care home staff and community nurses. J Appl Gerontol.

[15] Kupeli N (2018). What are the barriers to integration of palliative care for those at the advanced stages of dementia living in care homes in the UK? Health Care Professional Perspective. Dementia.

[16] Mitchell SL, Kiely DK, Hamel MB (2004). Dying with advanced dementia in the nursing home. Arch Intern Med.

[17] Miranda R (2019). Palliative care for people with dementia living at home: a systematic review of interventions. Palliat Med.

[18] Leniz J (2019). Understanding which people with dementia are at risk of inappropriate care and avoidable transitions to hospital near the end-of-life: a retrospective cohort study. Age Ageing.

[19] Social Care Institute for Excellence. Commissioning home care for older people. 2014 [cited 2017 23 June]; Available from: http://www.scie.org.uk/publications/guides/guide54/files/guide54.pdf.

[20] Alzheimer’s Disease International. Global estimates of informal care. In: Prince M, Gauthier S, Wimo A, editors. : Alzheimer’s Disease International; 2018. –London.

[21] Langa KM (2001). National estimates of the quantity and cost of informal caregiving for the elderly with dementia. J Gen Intern Med.

[22] Li Q, Loke AY (2013). The positive aspects of caregiving for cancer patients: a critical review of the literature and directions for future research. Psychooncology.

[23] Moore KJ (2017). Experiences of end of life amongst family carers of people with advanced dementia: longitudinal cohort study with mixed methods. BMC Geriatr.

[24] Muders P, et al. Support for families of patients dying with dementia: a qualitative analysis of bereaved family members' experiences and suggestions. Palliat Support Care. 2014:1–8. 10.1017/S1478951513001107 24524412

[25] Thuné-Boyle I, Wilcock J, Iliffe S (2013). Communicating with carers about dementia. Int J Geriatr Psychiatry.

[26] Davies N (2016). Family caregivers’ conceptualisation of quality end-of-life care for people with dementia: a qualitative study. Palliat Med.

[27] Saini G (2016). An ethnographic study of strategies to support discussions with family members on end-of-life care for people with advanced dementia in nursing homes. BMC Palliat Care.

[28] Davies N (2014). Quality end-of-life care for dementia: what have family carers told us so far? A narrative synthesis. Palliat Med.

[29] Crowther J (2013). Compassion in healthcare - lessons from a qualitative study of the end of life care of people with dementia. J R Soc Med.

[30] van der Steen JT (2017). Palliative care for people with dementia in the terminal phase: a mixed-methods qualitative study to inform service development. BMC Palliat Care..

[31] Kupeli N (2016). Context, mechanisms and outcomes in end of life care for people with advanced dementia. BMC Palliat Care..

[32] Pawson R, Tilley N (1997). Realistic Evaluation.

[33] Pawson R. The science of evaluation: a realist manifesto: Sage; 2013.

[34] Jones L (2016). Development of a model for integrated care at the end of life in advanced dementia: a whole systems UK-wide approach. Palliat Med.

[35] Bunn F (2018). Improving living and dying for people with advanced dementia living in care homes: a realist review of Namaste care and other multisensory interventions. BMC Geriatr.

[36] Reisberg B (1988). Functional assessment staging (FAST). Psychopharmacol Bull.

[37] Jones L (2012). CoMPASs: IOn programme (care of memory problems in advanced stages of dementia: improving our knowledge): protocol for a mixed methods study. BMJ Open.

[38] Sampson EL (2018). Living and dying with advanced dementia: a prospective cohort study of symptoms, service use and care at the end of life. Palliat Med.

[39] Braun V, Clarke V (2006). Using thematic analysis in psychology. Qual Res Psychol.

[40] Comondore VR (2009). Quality of care in for-profit and not-for-profit nursing homes: systematic review and meta-analysis. BMJ..

[41] Raymond M (2014). Palliative care services for people with dementia: a synthesis of the literature reporting the views and experiences of professionals and family carers. Dementia..

[42] Handley M, Bunn F, Goodman C (2017). Dementia-friendly interventions to improve the care of people living with dementia admitted to hospitals: a realist review. BMJ Open.

[43] Arcand M (2015). End-of-life issues in advanced dementia. Can Fam Physician.

[44] Robison J (2007). Partners in caregiving in a special care environment: cooperative communication between staff and families on dementia units. The Gerontologist.

[45] Dalmer NK. ‘Add info and stir’: an institutional ethnographic scoping review of family care-givers’ information work. Ageing Soc. 2018:1–27.

[46] Cahill S (2018). Dementia and human rights.

[47] Moore KJ (2017). Implementing the compassion intervention, a model for integrated care for people with advanced dementia towards the end of life in nursing homes: a naturalistic feasibility study. BMJ Open.

